# Volatile Organic Compound from *Trichoderma asperelloides* TSU1: Impact on Plant Pathogenic Fungi

**DOI:** 10.3390/jof7030187

**Published:** 2021-03-05

**Authors:** On-Uma Ruangwong, Prisana Wonglom, Nakarin Suwannarach, Jaturong Kumla, Narit Thaochan, Putarak Chomnunti, Kitsada Pitija, Anurag Sunpapao

**Affiliations:** 1 Department of Entomology and Plant Pathology, Faculty of Agriculture, Chiang Mai University, Chiang Mai 50200, Thailand; on-uma.r@cmu.ac.th; 2 Innovative Agriculture Research Center, Faculty of Agriculture, Chiang Mai University, Chiang Mai 50200, Thailand; 3 Faculty of Technology and Community Development, Phatthalung Campus, Thaksin University, 222 Moo 2, Ban Phrao Sub-District, Pa Payom District, Phattalung 93110, Thailand; prisana.w@tsu.ac.th; 4 Research Center of Microbial Diversity and Sustainable Utilization, Chiang Mai University, Chiang Mai 50200, Thailand; suwan.462@gmail.com (N.S.); Jaturong_yai@hotmail.com (J.K.); 5 Department of Biology, Faculty of Science, Chiang Mai University, Chiang Mai 50200, Thailand; 6 Agricultural Innovation and Management Division (Pest Management), Faculty of Natural Resources, Prince of Songkla University, Hatyai, Songkhla 90110, Thailand; narit.t@psu.ac.th; 7 School of Science, Mae Fah Luang University, Chiang Rai 57100, Thailand; putarak.cho@mfu.ac.th; 8 Perkin Elmer Co. Ltd., 290 Soi 17, Rama 9 Rd., Bangkapi, Huay Kwang, Bangkok 10310, Thailand; Kitsada.pitija@perkinelmer.com

**Keywords:** soil fungi, *Trichoderma*, VOCs, plant pathogen, antifungal activity

## Abstract

Soil microorganisms are well studied for their beneficial effects on plant growth and their impact on biocontrol agents. The production of volatile antifungal compounds emitted from soil fungi is considered to be an effective ability that can be applied in biofumigants in the control of plant diseases. A soil fungus, *Trichoderma asperelloides* TSU1, was isolated from flamingo flower cultivated soil and identified on the basis of the morphology and molecular analysis of the internal transcribed spacer (ITS), *rpb2*, and *tef1-α* genes. To test *T. asperelloides* TSU1-produced volatile organic compounds (VOCs) with antifungal activity, the sealed plate method was used. The VOCs of *T. asperelloides* TSU1 inhibited the mycelial growth of fungal pathogens that were recently reported as emerging diseases in Thailand, namely, *Corynespora cassiicola*, *Fusarium incarnatum*, *Neopestalotiopsis clavispora*, *N. cubana*, and *Sclerotium rolfsii,* with a percentage inhibition range of 38.88–68.33%. Solid-phase microextraction (SPME) was applied to trap VOCs from *T. asperelloides* TSU1 and tentatively identify them through gas chromatography–mass spectrometry (GC/MS). A total of 17 compounds were detected in the VOCs of *T. asperelloides* TSU1, and the dominant compounds were identified as fluoro(trinitro)methane (18.192% peak area) and 2-phenylethanol (9.803% peak area). Interestingly, the commercial 2-phenyethanol showed antifungal activity against fungal pathogens that were similar to the VOCs of *T. asperelloides* TSU1 by bioassay. On the basis of our study’s results, *T. asperelloides* TSU1 isolated from soil displayed antifungal abilities via the production of VOCs responsible for restricting pathogen growth.

## 1. Introduction

Soil is an important source of beneficial microorganisms. Multiple soil fungi participate in the decomposition of organic matter to deliver plant nutrients [1]. They play an important role in plant protection against pathogens as biocontrol agents that influence soil health [2]. *Trichoderma* is a genus of filamentous ascomycete fungi that are frequently isolated from soil or rhizosphere soil in tropical areas containing approximately 10^1^–10^3^ culturable propagules per gram [3,4]. *Trichoderma* is the most important genus of beneficial microorganisms in agricultural soil, and it has been widely used in crop-plant production [5]. It is used as a natural-decomposition agent [6], plant-growth promoter [7], and as a biocontrol agent of plant diseases [8,9] and bioremediation [10].

One of the most dominant abilities of *Trichoderma* species is as a biocontrol agent against plant diseases. Several *Trichoderma* strains were widely studied due to their capacity to compete for nutrients and space [11,12], parasitize other fungi [13,14], enact antibiosis by producing secondary metabolites or antimicrobial compounds [15,16], induce defense responses in plants [17,18], and promote plant growth [15,19]. The key to the success of *Trichoderma* species in the soil is their fast growth and high reproductive ability, aiding them in surviving under unfavorable conditions, and their ability to utilize nutrients and antagonize plant pathogenic fungi [20,21].

Producing volatile organic compounds (VOCs) is considered to be an effective antibiosis mechanism of *Trichoderma* species. *Trichoderma* species produce and emit valuable volatiles associated with antimicrobial ability, induce defense responses in plants, and promote plant growth [15,16]. VOCs released by several species of microorganisms directly inhibit the growth of pathogens or cause abnormal changes of pathogens, resulting in the weakness of pathogens interacting with plant hosts [22,23]. For instance, *T. spirale* T76-1 was reported to produce VOCs to restrict the fungal growth of *Corynespora cassiicola* and *Curvularia aeria,* the leaf-spot pathogens in lettuce [12]. Wonglom et al. [24] also reported that *Trichoderma* sp. T76-12/2 produces VOCs to inhibit the growth of *Sclerotium rolfsii*.

The capacity to produce beneficial compounds, especially the VOCs, by *Trichoderma* species is particularly interesting. Some *Trichoderma* strains produce valuable VOCs with multifaceted mechanisms involved in biological control, inducing defense responses and promoting plant growth [12,15,16]. The antifungal ability of VOCs released by *Trichoderma* is highly interesting because effective VOCs can be applied as biofumigants in controlling plant diseases and pests. Therefore, the screening of novel volatile-producing *Trichoderma* strains is still being conducted. As soil is the main source of beneficial microorganisms, this study characterizes the antifungal activity of VOCs released from *Trichoderma* that were isolated from soil. The isolation of *Trichoderma*, the identification of *Trichoderma* by morphology and molecular properties, and VOC bioassay against plant fungal pathogens were conducted.

## 2. Materials and Methods

### 2.1. Isolation and Morphological Identification

*Trichoderma* species strain TSU1 was isolated from the soil of the flamingo flower (*Anthurium*) cultivation farm, Nakhon Si Thammarat province, southern Thailand, by the soil-dilution pour-plate method. A primary screening revealed the competition ability of *Trichoderma* sp. TSU1 against plant fungal pathogens. This strain was subjected to morphological and molecular identification. *Trichoderma* sp. TSU1 was cultured on potato dextrose agar (PDA); macroscopic and microscopic features were observed by a Leica S8AP0 stereomicroscope (Leica Microsystems, Wetzlar, Germany) and a Leica DM750 compound microscope (Leica Microsystems, Wetzlar, Germany), respectively.

### 2.2. Pathogen Sources

Plant pathogens recently causing emerging diseases in Thailand were obtained from Culture Collection of Pest Management, Faculty of Natural Resources, Prince of Songkla University; details are provided in Table 1. Airborne pathogens were cultured on PDA for three days before being subjected to bioassay: *Corynespora cassiicola*, causing leaf spots on lettuce [25]; *Neopestalotiopsis clavispora*, causing flower blight on anthurium [26]; and *Neopestalotiopsis cubana*, causing leaf fall in rubber trees [27]. The soilborne pathogen was *Sclerotium rolfsii*, causing stem rot in Jerusalem artichoke. The postharvest pathogen was *Fusarium incarnatum,* causing fruit rot in muskmelon [28].

### 2.3. Molecular Identification

*Trichoderma* sp. TSU1 was cultured on PDA for 3 days and subjected to DNA extraction by DNA Extraction Mini Kit (FAVORGEN, Ping-Tung, Taiwan) following the manufacturer’s protocol. A polymerase chain reaction (PCR) was performed in 20 µL volume containing 1.0 µL DNA template, 1.0 µL of each primer, 10.0 µL 2× Quick Taq^®^ HS DyeMix (TOYOBO, Saitama, Japan), and 7 µL deionized water. PCR amplification of internal transcribed spacer (ITS), part of RNA polymerase 2 (*rpb2*), and translation elongation factor 1-α (*tef1-α*) genes were amplified by using ITS1/ITS4 [29], fRPB2-5F/fRPB2-7cr [30], and Tef1-728F [31]/Tef1-986R [32] primer pairs, respectively, in the following thermal conditions: 94 °C for 2 min, followed by 35 cycles of 94 °C for 2 min, annealing at a temperature dependent on the amplified gene (Table 2) for 60 s and 72 °C for 1 min, and a final 72 °C for 10 min in a peqSTAR thermal cycler (PEQLAB Ltd., Fareham, UK). PCR products were observed on 1% agarose gels stained with ethidium bromide under UV light. PCR products were purified using a PCR clean-up Gel Extraction NucleoSpin^®^ Gel and PCR Clean-Up Kit (Macherey-Nagel, Düren, Germany). The purified PCR products were directly sequenced. Sanger sequencing was carried out by 1st Base Company (Kembangan, Selangor, Malaysia) using the PCR primers mentioned above. Sequences were used to query the GenBank gene sequence database via BLAST (http://blast.ddbj.nig.ac.jp/top-e.html, accessed on 6 December 2020).

Multiple-sequence alignment was carried out using MUSCLE [33]. A phylogenetic tree was constructed using maximum-likelihood (ML) and Bayesian inference (BI) methods. The best substitution models for ML and BI analyses were estimated by the Akaike information criterion (AIC) in jModeltest v2.1.10 [34]. ML analysis was carried out on RAxML v7.0.3 under the GTR + I + G model with 1000 bootstrap replications [35,36]. BI analysis was conducted with MrBayes v3.2.6 [37] to evaluate the posterior probabilities (PPs) by Markov chain Monte Carlo (MCMC) sampling. Markov chains were run for 1 million generations; trees were sampled every 100th generation, and 10,000 trees were obtained. The first 2000 trees representing the burning phase of the analyses were discarded. The remaining 8000 trees were then used to calculate PP in the majority-rule consensus tree. Bootstrap support (BS) and PP values greater than or equal to 70% and 0.95 of each branch, respectively, were considered to be significantly supported [38,39].

### 2.4. Sealed Plate Method

To test the effect of volatiles released from *Trichoderma* TSU1 in suppressing the fungal growth of plant pathogens, the sealed plate method was used [40] with some modifications. *Trichoderma* TSU1 was grown on PDA in 8 cm Petri dishes (Citotest, Jiangsu, Haimen, China) for 3 days. A mycelial plug (0.5 cm in diameter) was cut from the culture plate, inserted centrally, and the lid of each Petri dish was removed. The bottom plate containing PDA inoculated with tested fungi was replaced, and the two bottom plates were then sealed together with Parafilm. For the control, the bottom plate with PDA alone was replaced, instead of the tested fungi, in the same manner. The experiment was conducted in five replicates and repeated twice. Tested plates were incubated at ambient temperature (28 ± 2 °C) for 3 days. Colony diameters of plant pathogens were measured and converted into the percentage of inhibition through the following formula

Percentage inhibition (%) = [(D*c* − D*t*)/D*c*] × 100 (1)

where D*c* is the mycelial growth of the plant pathogen on the control plate, and D*t* is the mycelial growth of the plant pathogen on the tested plate.

### 2.5. SPME/GC/MS Analysis

To determine the volatile compounds released from *Trichoderma* TSU1, gas chromatography–mass spectrophotometry (GC/MS) was conducted [15,16]. The TSU1 strain was cultured in PDA in a 20 mL chromatography vial, 20 mm in diameter (PerkinElmer, Waltham, MA, USA), and incubated at ambient temperature (28 ± 2 °C) for 7 days. Solid-phase microextraction (SPME) was performed to extract the volatile organic compounds (VOCs) emitted by TSU1. The SPME fiber (DVB/CAR/PDMS fiber) was exposed to vapors above the TSU1 culture for 30 min and inserted into the injection port of an SQ8 gas chromatograph (PerkinElmer, Waltham, MA, USA), equipped with a DB-Wax capillary column (30 m × 0.25 mm i.d., 0.25 μm film thickness). The oven temperature was initially maintained at 40 °C and then increased to 230 °C at a rate of 7 °C min^−1^. The injector temperature was 230 °C. The carrier gas was ultrahigh-purity helium with an initial column head pressure of 60 kPa at a flow rate of 1 mL min^–1^. Electron-impact (EI) mass spectra were collected at 70 eV ionization voltage over the *m z*^–1^ range of 29–550. Ion-source and quadrupole temperatures were both set at 200 °C. Volatiles emitted by TUS1 were tentatively identified on the basis of computer searches dictated by The National Institute of Standards and Technology (NIST) Mass Spectral Library Search Chromatogram.

### 2.6. Effect of Commercial Volatile Compounds on Fungal Growth

To test the effect of commercial volatile compounds against plant pathogens, the sealed plate method was conducted as described in Section 2.4. The commercial volatile compound 2-phenylethanol (2-PE) was purchased from Sigma-Aldrich (St. Louis, MO, USA), dissolved in 95% ethanol, and dilution was adjusted to 10^–1^, 10^–2^, or 10^–3^. The solution of 2-PE was applied on a sterile cotton pad (20 µL) and subjected to the method of Wonglom et al. [15]. The application of 95% ethanol served as the negative control. Bioassay plates were incubated at ambient temperature for seven days. Each treatment was composed of five replicates, and the experiment was repeated twice. The colony diameters of plant pathogens were measured, and the percentage inhibition was calculated as described in Section 2.3.

### 2.7. Statistical Analysis

Results on fungal inhibition were subjected to one-way ANOVA. Statistically significant differences among treated samples were determined by Tukey’s test.

## 3. Results

### 3.1. Taxonomy

*Trichoderma asperelloides* (TSU1) Samuels, *Mycologia* 102(4): 961 (2010).

**Figure 1:**
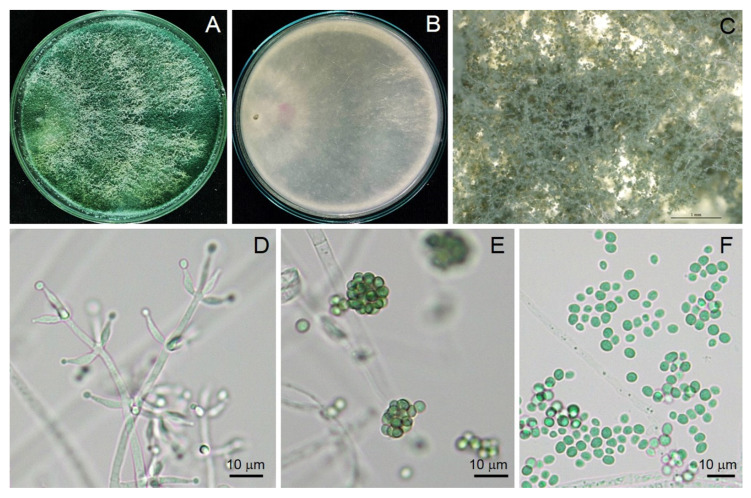
Morphological characteristics of *Trichoderma asperelloides* strain TSU1 colony on potato dextrose agar (PDA) in (**A**) top and (**B**) bottom view; (**C**) colony observed under stereomicroscope; (**D**) detail of conidiophores and phialides; (**E**) mass of conidia and (**F**) conidia.

Conidia germinated on PDA within 24 h, reaching 8.5 mm diameter in four days at 28 ± 2 °C. Colonies on PDA with white, downy mycelia turned pale green and dark green after a few days (Figure 1). Conidiophores branched, produced in pustules, and secondary branches arose. The secondary branches tended to be paired and asymmetric, terminating in a single phialide or a whorl of two to three divergent phialides. Phialides were 5.43–11.76 μm long, 1.50–3.44 μm wide at the base ($\bar{x}$ = 7.74 × 2.22 μm, *n* = 30), and ampulliform. Conidia were 2.48–4.59 × 2.13–3.93 μm ($\bar{x}$ = 3.30 × 3.02 μm, *n* = 30), dark green, and subglobose (Figure 1).

**Figure 2:**
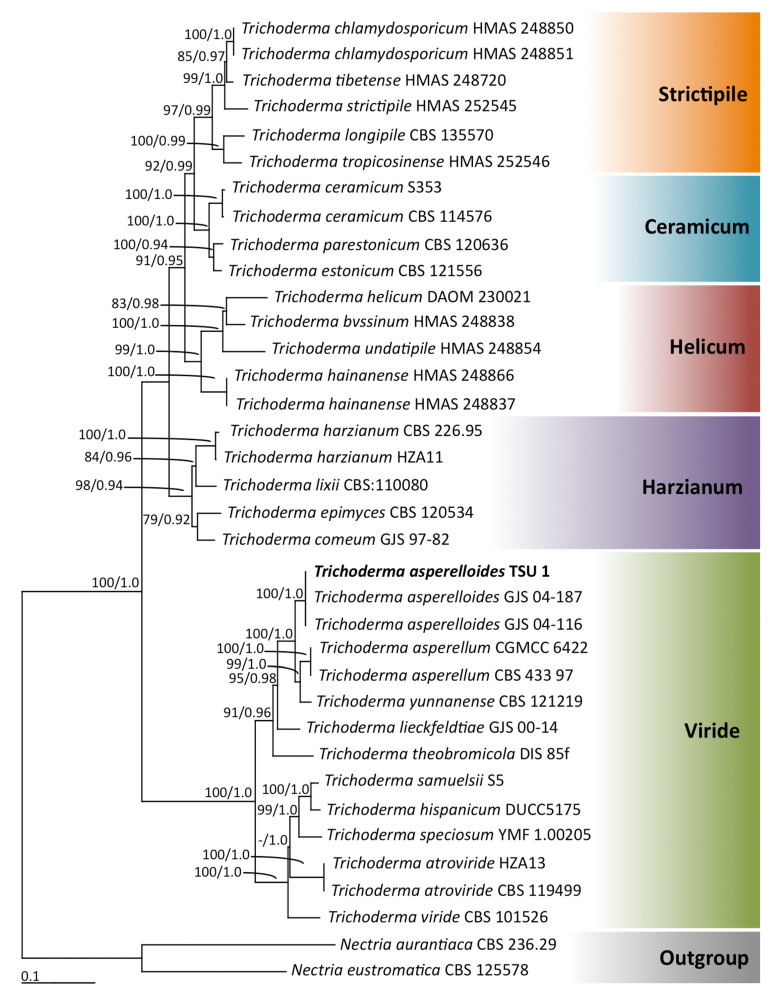
Phylogram derived from maximum-likelihood analysis of combined ITS, *rbp*2, and *tef1-α* genes of 36 taxa of *Trichoderma*. Sequences of *Nectria aurantiaca* and *N. eustromatica* used as the outgroup. Numbers above branches represent (**left**) maximum-likelihood bootstrap percentages and (**right**) Bayesian posterior probabilities. Bootstrap values ≥70% and Bayesian posterior probabilities ≥0.90 shown. Scale bar represents 10 substitutions per nucleotide position. Sequences of fungal species obtained in this study are in bold.

Phylogenetic analyses: Multiple genes were amplified through PCR, and their nucleotide sequences were analyzed. A BLAST search revealed that the sequences of ITS, *rpb2,* and *tef1-α* genes matched 100% with *T. asperelloides*. Therefore, our isolation strain TSU1 was identified as *T. asperelloides* [32,41]. The partial ITS, *rbp*2, and *tef1-α* sequences of this fungal isolate were deposited in GenBank as MW504989, MW546917, and MW546916, respectively. The combined ITS, *rbp*2, and *tef1-α* sequence dataset consisted of 36 taxa, and the aligned dataset comprised 2332 characters including gaps (ITS: 1–650, *rbp*2: 651–1745, and *tef*1-α: 1746–2436). ML analysis of the combined ITS, *rbp*2, and *tef*1-α sequences was based on the estimated parameters of the GTR + I + G model, and the proportion of the invariable sites and gamma shape parameters were 0.3730 and 0.8260, respectively. Additionally, the tree with a log-likelihood of –12,412.6914 was built after 1000 bootstrap replications. The average standard deviation of the split frequencies of BI analysis was 0.00536. The phylograms of ML and BI analyses were similar in topology. Therefore, we only present the phylogram obtained from ML analysis (Figure 2). Five main clades—namely, Ceramicum, Harzianum, Helicum, Strictipile, and Viride—were assigned according to the findings of previous phylogenetic studies [42,43]. The results indicated that fungal isolate TSU1, placed in the monophyletic clade of *T*. *asperelloides,* was within the Viride clade with high supporting values (BS 100% and BP 1.0). *T*. *asperelloides* formed a sister taxon to *T. asperellum* and *T*. *yunnanense*. Therefore, the isolated TSU1 was identified as *T*. *asperelloides* on the basis of morphological, physiological, biochemical, and molecular characteristics.

### 3.2. Trichoderma asperelloides TSU1 Released VOCs against Fungal Pathogens

**Figure 3:**
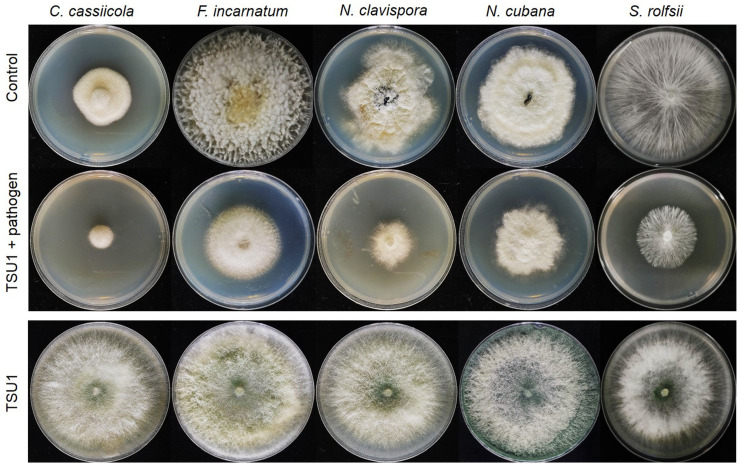
Sealed plate method for the analysis of the activity of volatile organic compounds (VOCs) released from *Trichoderma asperelloides* TSU1 against fungal pathogens *Corynespora cassiicola*, *Fusarium incarnatum*, *Neopestalotiopsis clavispora*, *N. cubana*, and *Sclerotium rolfsii*.

**Figure 4:**
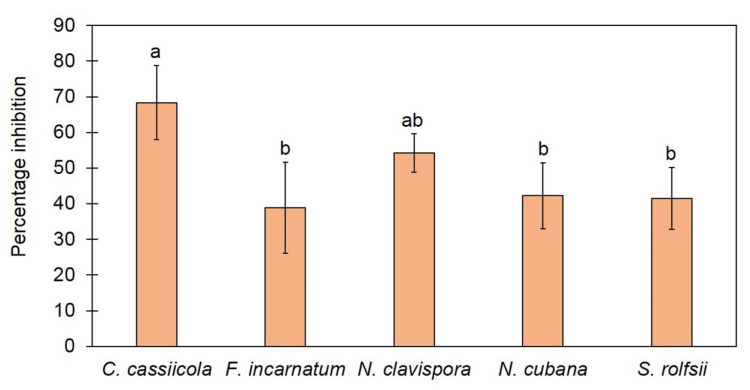
Percentage inhibition of VOCs emitted from *Trichoderma asperelloides* TSU1 against fungal pathogens by sealed plate method. Different letters indicate statistically significant differences among treatments (*p* < 0.05) using Tukey’s test.

We tested the effect of VOCs released from *T. asperelloides* TSU1 against five fungal pathogens, namely, *C. cassiicola*, *F. incarnatum*, *N. clavispora*, *N. cubana,* and *S. rolfsii,* through the sealed plate method. The results show that *T. asperelloides* TSU1 released VOCs with antifungal abilities for the inhibition of fungal pathogen growth (Figure 3). Fungal growth in the tested plates was significantly smaller than that in the control plates (*p* < 0.05). The percentage inhibition of VOCs emitted by *T. asperelloides* TSU1 against fungal pathogens was in the range of 38.88–68.33% (Figure 4). The percentage inhibition of VOCs against *C. cassiicola*, *F. incarnatum*, *N. clavispora*, *N. cubana,* and *S. rolfsii* was 68.33%, 38.88%, 54.22%, 42.22%, and 41.19%, respectively (Figure 4). VOCs emitted from *T. asperelloides* TSU1 effectively inhibited the fungal growth of *C. cassiicola* causing lettuce leaf spot (68.33%), followed by *N. clavispora* causing anthurium flower blight (54.22%).

### 3.3. Identification of VOCs through SPME/GC/MS

**Figure 5:**
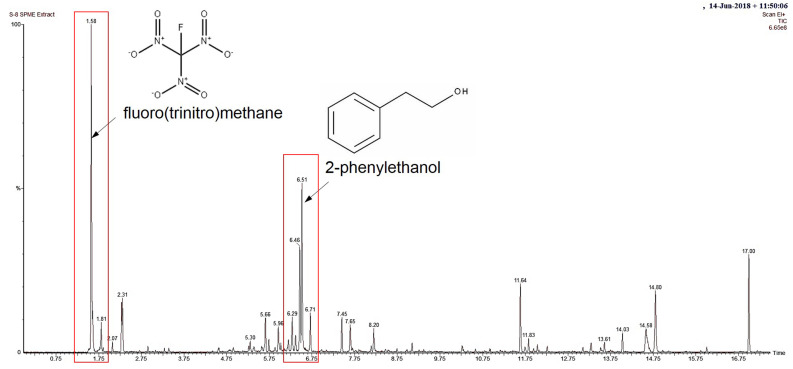
Total ion chromatogram of volatile compounds identified from *T. asperelloides* TSU1 through gas chromatography–mass spectrometry (GC/MS) analysis, and peaks at 1.58 min and 6.51 min tentatively identified as fluoro(trinitro)methane and 2-phenylethanol (red rectangles), respectively, and their structures.

The results above show that VOCs released from *T. asperelloides* TSU1 inhibited the growth of fungal pathogens, suggesting antifungal ability. The identification of the volatiles of *T. asperelloides* TSU1 was conducted through SPME/GC/MS. A total of 17 compounds with percentages matching >70% were tentatively identified using an NIST library search (Table 3). The detected compounds in *T. asperelloides* TSU1 contained carbon numbers ranging from C_1_ to C_21_, classified as members of the following compound classes: alcohol, fatty acid, pyran, and terpene. The most dominant volatile found in this study was fluoro(trinitro)methane, followed by 2-phenylethanol (2-PE), with percentage peak areas of 18.192% and 9.803%, respectively (Figure 5). According to previous studies, only 2-PE was shown to suppress fungal growth. Figure 5 shows the mass spectrum of major compounds and the structure of 2-PE. No major peaks were observed in PDA alone, which served as the control group.

### 3.4. Effect of 2-PE on Fungal Growth

**Figure 6:**
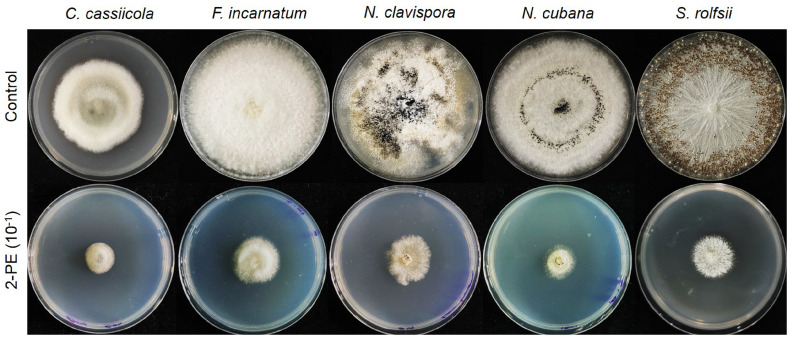
Sealed plate method for the analysis of the effects of commercial 2-PE at 10^−1^ dilution against fungal pathogens *Corynespora cassiicola*, *Fusarium incarnatum*, *Neopestalotiopsis clavispora*, *N. cubana,* and *Sclerotium rolfsii*.

**Figure 7:**
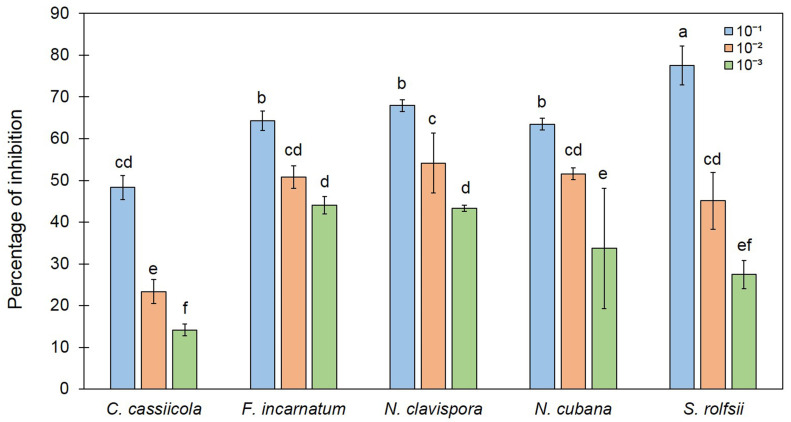
Percentage inhibition of commercial 2-PE at different dilution levels (10^−1^, 10^−2^, 10^−3^) against fungal pathogens by sealed plate method. Different letters indicate statistically significant differences among treatments (*p* < 0.05) using Tukey’s test.

We found 2-PE to be a major peak by SPME/GC/MS analysis (Figure 5), and it was reported as an antifungal compound [44]. 2-Phenylethanol is a primary alcohol that is ethanol-substituted by a phenyl group at position 2. It has a role as a fragrance, a *Saccharomyces cerevisiae* metabolite, a plant metabolite, an *Aspergillus metabolite*, and a plant growth retardant. It is a primary alcohol and a benzene, and is an antimicrobial, antiseptic, and disinfectant that is also used as an aromatic essence and preservative in pharmaceutics and perfumery. In this study, commercial 2-PE was diluted at concentrations of 10^−1^, 10^−2^, and 10^−3^ and subjected to the sealed plate method to test its antifungal activity against plant fungal pathogens. The results show that the fungal colonies of the tested plates were smaller than that of the control, similar to the result of VOCs from *T. asperelloides* TSU1 (Figure 6). The high-percentage inhibition was related to the high concentration. At 10^–1^ dilution, the commercial 2-PE inhibited fungal growth with a percentage inhibition ranging from 48.33% to 77.51% (Figure 7). The percentage inhibition of 2-PE against *C. cassiicola*, *F. incarnatum*, *N. clavispora*, *N. cubana,* and *S. rolfsii* was 48.33%, 64.28%, 67.91%, 63.49%, and 77.51%, respectively (Figure 7).

## 4. Discussion

In this study, a soil fungus was isolated and identified on the basis of morphology and molecular techniques, and antifungal activity against plant diseases was examined. The isolated fungus was identified as *T. asperelloides* TSU1 (Figure 1 and Figure 2). This isolate was documented as being capable of emitting 17 VOCs, and results indicate that its VOCs were responsible for the in vitro inhibition of fungal growth for some airborne, soilborne, and postharvest pathogens (Figure 3 and Figure 4). The antifungal activity of the VOCs from this isolate was comparable to the adverse activity of commercial 2-PE against the same group of tested fungal plant pathogens. Interestingly, the commercial 2-phenyethanol showed antifungal activity against fungal pathogens that was similar to the VOCs of *T. asperelloides* TSU1 by bioassay.

Low-molecular-weight volatile phase compounds produced by growing *Trichoderma* species are chemically diverse and possess antimicrobial properties. The antifungal activity of *T. asperelloides* TSU1 VOCs that suppress the growth of fungal pathogens is correlated with the presence of 2-PE. The volatile eight-carbon compound 2-PE is an important element that imparts flavor and taste in food with a rose-like aroma. Interestingly, 2-PE showed antifungal ability against plant diseases. For instance, Liu et al. [45] applied 2-pehynylethanol isolated from *Kloeckera apiculata* to control the *Penicillium* mold of citrus. Here, *T. asperelloides* TSU1 produced VOCs that contained 2-PE, and effectively inhibited the growth of fungal pathogens (Figure 6 and Figure 7). Therefore, the ability to produce antifungal compounds of *T. asperelloides* TSU1 may control several plant diseases.

The ability to produce VOCs by endophytic fungus *T. asperelloides* strain PSU-P1 was recently reported to inhibit fungal pathogens, induce a defense response, and promote plant growth in *Arabidopsis thaliana* [16]. Our result agrees with those reporting that *T. asperelloides* TSU1 produces VOCs to inhibit the growth of plant pathogens (Figure 3). The pattern of the antifungal ability of VOCs and commercial 2-PE is seemed to be differed (Figure 4 and Figure 7). The effects of the volatiles from the fungus were more effective on *C. cassiicola* than on *S. rolfsii* (Figure 4), while the results in Figure 7 show a lower effect on *C. cassiicola* and a higher effect on *S. rolfsii*. This phenomenon may be explained by how volatiles of *Trichoderma* were produced and released in a complex mixture containing 17 compounds; some compounds might work synergistically to inhibit fungal growth with more effective inhibition than with single compounds [15]. For a commercial volatile, pure 2-PE was used, which may affect the different inhibitions against plant pathogens. Furthermore, fungi produce a wide range of different types of hyphae; therefore, the effect of the volatile mixture and the single compound may also differ, resulting in different inhibitory activities in the bioassay.

Phoka et al. [16] recently revealed that *T. asperelloides* PSU-P1, an endophytic fungus, released major volatile compounds (namely, 2-methyl-1-butanol and 6-pentyl-2H-pyran-2-one) responsible for antifungal ability. Our results demonstrated that a different strain of the same species, *T. asperelloides* (TSU1), released 2-PE. This study provides evidence that VOCs emitted from *T. asperelloides* TSU1 have antifungal effects against some plant pathogens and could be used as a suitable alternative to synthetic fungicides. Biofumigation by microorganisms is an alternative way to control postharvest diseases that is environmentally friendly and less toxic to human health.

## 5. Conclusions

A new strain of *T. asperelloides* TSU1 isolated from flamingo flower cultivated soil showed antifungal ability against fungal pathogens causing emerging diseases in Thailand via the production of VOCs. These findings suggest that *T. asperelloides* TSU1 produces effective VOCs that may be used to control fungal pathogens. To apply VOCs released by *T. asperelloides* as a biofumigant for controlling plant diseases, especially postharvest diseases, more experiments are needed.

## Figures and Tables

**Table 1:** List of fungal pathogens used for bioassay in this study.

Pathogen	Disease	Host	Sources
*Corynespora cassiicola*	Leaf spot	*Lactuca sativa*	[25]
*Fusarium incarnatum*	Fruit rot	*Cucumis melo*	[28]
*Neopestalotiopsis clavispora*	Flower blight	*Anthurium andraeanum*	[26]
*N. cubana*	Leaf fall	*Hevea brasiliensis*	[27]
*Sclerotium rolfsii*	Stem rot	*Helianthus tuberosus*	CCPM *

* Culture Collection of Pest Management (PSU-HT01), Faculty of Natural Resources, Prince of Songkla University.

**Table 2:** Details of primers and annealing temperatures used for the amplification of gene targets in this study.

Region	Primer	Orientation	Annealing Temperature (°C)	Reference
ITS *	ITS5	Forward	50	[29]
	ITS4	Reverse	50	[29]
*rpb2*	fRPB2-5F	Forward	54	[30]
	fRPB2-7cr	Reverse	54	[30]
*tef1-α*	Tef1-728F	Forward	52	[31]
	Tef1-986R	Reverse	52	[32]

* ITS = internal transcribed spacer; *rpb2* = RNA polymerase II; *tef1-α* = translation elongation factor 1-α.

**Table 3:** International Union of Pure and Applied Chemistry (IUPAC) names of volatile compounds produced by *Trichoderma* TSU1 identified through solid-phase microextraction (SPME)/GC/MS analysis.

Retention Time	IUPAC Name	Formula	Percentage Match (%)	Percentage Area (%)
1.575	Fluoro(trinitro)methane	CFN_3_O_6_	95	18.192
2.309	Azetidine	C_3_H_7_N	87.1	2.628
5.303	1-Methyl-2-propan-2-ylbenzene	C_10_H_14_	81.9	0.675
5.656	3,7-Dimethylundecane	C_13_H_28_	82.2	2.182
6.287	5,7-Dimethylundecane	C_13_H_28_	84.4	2.215
6.464	4-Methyl-2-(2-methylprop-1-enyl)oxane	C_10_H_18_O	85.1	5.639
6.514	2-Phenylethanol	C_8_H_10_O	92.1	9.803
6.712	(2*R*,4*R*)-4-Methyl-2-(2-methylprop-1-enyl)oxane	C_10_H_18_O	91.6	2.296
7.453	(2,2-Dimethylcyclopentyl)cyclohexane	C_13_H_24_	68.6	1.989
8.201	1,3-Benzothiazole	C_7_H_5_NS	82.3	1.506
11.639	1-Methyl-4-(6-methylhept-5-en-2-yl)cyclohexa-1,4-diene	C_15_H_24_	82	4.386
11.831	7,11-Dimethyl-3-methylidenedodeca-1,6,10-triene	C_15_H_24_	76.1	0.811
13.297	4,7-Dimethyl-1-propan-2-yl-2,3,4,5,6,8*a*-hexahydro-1*H*-naphthalen-4*a*-ol	C_15_H_26_O	74.3	0.701
14.033	Cyclohexylmethyl hexyl sulfite	C_13_H_26_O_3_S	77.8	1.418
14.578	Methyl icosa-11,14-dienoate	C_21_H_38_O_2_	82.5	3.708
14.804	Ethyl (*E*)-octadec-9-enoate	C_20_H_38_O_2_	82.5	5.449
16.998	Ethyl hexadecanoate	C_18_H_36_O_2_	84.4	6.003

## Data Availability

Not applicable.
